# Functional role of SGK3 in PI3K/Pten driven liver tumor development

**DOI:** 10.1186/s12885-019-5551-2

**Published:** 2019-04-11

**Authors:** Hui Cao, Zhong Xu, Jingxiao Wang, Antonio Cigliano, Maria G. Pilo, Silvia Ribback, Shu Zhang, Yu Qiao, Li Che, Rosa M. Pascale, Diego F. Calvisi, Xin Chen

**Affiliations:** 1 Department of Oncology, Guizhou Provincial People’s Hospital, Medical College of Guizhou University, Guiyang, People’s Republic of China; 2 Department of Bioengineering and Therapeutic Sciences and Liver Center, University of California, UCSF, 513 Parnassus Ave, San Francisco, CA 94143 USA; 3 Department of Gastroenterology, Guizhou Provincial People’s Hospital, Medical College of Guizhou University, Guiyang, People’s Republic of China; 4 Second Clinical Medical School, Beijing University of Chinese Medicine, Beijing, People’s Republic of China; 5 National Institute of Gastroenterology “S. de Bellis”, Research Hospital, Castellana Grotte, Italy; 6 Department of Clinical and Experimental Medicine, University of Sassari, via P. Manzella 4, 07100 Sassari, Italy; 7 Institute of Pathology, University of Greifswald, Greifswald, Germany; 8 Department of Oncology, Beijing Hospital, Beijing, People’s Republic of China

**Keywords:** Liver cancer, SGK3, PIK3CA mutants, c-Met, Pten, mTOR

## Abstract

**Background:**

Hepatocellular carcinoma (HCC) is a leading cause of cancer related deaths worldwide. The PI3K cascade is one of the major signaling pathways underlying HCC development and progression. Activating mutations of PI3K catalytic subunit alpha (PIK3CA) and/or loss of Pten often occur in human HCCs. Serum and glucocorticoid kinase 3 (SGK3) belongs to the SGK family of AGK kinases and functions in parallel to AKT downstream of PI3K. Previous studies have shown that SGK3 may be the major kinase responsible for the oncogenic potential of PIK3CA helical domain mutants, such as PIK3CA(E545K), but not kinase domain mutants, such as PIK3CA(H1047R).

**Methods:**

We investigated the functional contribution of SGK3 in mediating activated PIK3CA mutant or loss of Pten induced HCC development using *Sgk3* knockout mice.

**Results:**

We found that ablation of *Sgk3* does not affect PIK3CA(H1047R) or PIK3CA(E545K) induced lipogenesis in the liver. Using PIK3CA(H1047R)/c-Met, PIK3CA(E545K)/c-Met, and sgPten/c-Met murine HCC models, we also demonstrated that deletion of *Sgk3* moderately delays PIK3CA(E545K)/c-Met driven HCC, while not affecting PIK3CA(H1047R)/c-Met or sgPten/c-Met HCC formation in mice. Similarly, in human HCC cell lines, silencing of SGK3 reduced PIK3CA(E545K) -but not PIK3CA(H1047R)- induced accelerated tumor cell proliferation.

**Conclusion:**

Altogether, our data suggest that SGK3 plays a role in transducing helical domain mutant PIK3CA signaling during liver tumor development.

**Electronic supplementary material:**

The online version of this article (10.1186/s12885-019-5551-2) contains supplementary material, which is available to authorized users.

## Background

Hepatocellular carcinoma (HCC) is one of the most common causes of death from cancer in the world [1]. Therapeutic approaches for the treatment of HCC at advanced stages are very limited. The multi-kinase inhibitors Sorafenib and Regorafenib have been approved for patients with advanced HCC over the last decade [2, 3]. However, they can extend patients’ survival by approximately three months. Thus, it is necessary to elucidate the molecular pathogenesis of HCC for alternative therapeutic strategies with improved potency.

As one of the most important intracellular signaling pathways, the Phosphoinositide-3-Kinase (PI3K)/mammalian target of rapamycin (mTOR) pathway is frequently altered in human cancers [4, 5], including HCC [6]. Extensive studies have shown that the PI3K/mTOR pathway plays a critical role in many cellular processes essential for tumorigenesis, including cell proliferation, growth, metabolism, angiogenesis, and survival [7]. In normal tissues, PI3K pathway is negatively regulated by the tumor suppressor protein phosphatase and tensin homolog (Pten) [8]. Deregulation of genes involved in the PI3K pathway, including mutations of the PI3K catalytic subunit alpha (PIK3CA) and loss of Pten, is frequently found in cancer [7]. Similar to other tumor types, recent genome-wide studies have revealed that both mutations of PIK3CA and deletion/downregulation of Pten occur in human HCCs [9].

PI3K functions via regulating the AGC family of kinases. Serum/glucocorticoid regulated kinase 3 (SGK3) is a protein kinase of the AGC family. It shares similar substrate specificity with AKT kinases and also functions as a downstream mediator of the PI3K cascade [10, 11]. As a target of PI3K, SGK3 has been found to be implicated in the regulation of several cellular processes such as cell growth, proliferation, survival, and migration [10, 12]. Mounting evidence indicates that SGK3 is involved in the development and progression of several cancers, including HCC, breast cancer, prostate cancer, and melanoma [11, 13–15]. In addition, Liu et al [16] recently reported that the SGK3 protooncogene plays a vital role in the expansion of liver cancer stem cells (CSCs) through the GSK3β/β-catenin signaling pathway.

PIK3CA mutations most frequently occur at two domains: the helical domain, such as E545K, and the kinase domain, such as H1047R [14]. Intriguingly, recent studies in breast cancer showed that cell lines harboring the E545K mutation have lower phosphorylated/activated (p-)AKT levels when compared with H1047R mutant cells [17, 18]. Also, the PIK3CA E545K mutant form promotes growth of breast cancer cells by activation of SGK3, but not AKT [18]. PIK3CA helical domain mutations are present in numerous cancer patients; thus, it has been hypothesized that targeting SGK3 may be an effective treatment option for tumors harboring PIK3CA helical domain mutations [19].

Recently, we reported the oncogenic cooperation between mutant forms of PIK3CA and c-Met pathways along liver carcinogenesis [20]. Specifically, hydrodynamic injection of PIK3CA(H1047R) or PIK3CA(E545K), together with c-Met, into the mouse liver promotes HCC formation within ~ 10 weeks post injection. These models are referred to as H1047R/c-Met and E545K/c-Met in this manuscript. In addition, we demonstrated that ablation of *Pten* by CRISPR-based technology (sgPten) synergizes with c-Met to promote HCC development (sgPten/c-Met) [21]. Importantly, we and others found that AKT2 is required for PIK3CA mutant or loss of Pten driven liver tumor development in mice [20, 22], suggesting that AKT2 is the major AGC kinases downstream of PI3K/Pten during hepatocarcinogenesis. However, whether SGK3 is required for HCC development, especially in the context of PIK3CA helical domain mutant, has not been investigated. In the present study, we utilized the preclinical models described above in the *Sgk3* knockout background to assess the importance of SGK3 signaling during liver tumorigenesis.

## Methods

### Constructs and reagents

The constructs used for mouse injection, including pT3-EF1α-PIK3CA(H1047R), pT3-EF1α-PIK3CA(E545K), pT3-EF1α-c-Met, PX330-sgPten, and pCMV/sleeping beauty transposase (pCMV/SB), were described previously [20, 21, 23–25]. pLenti-PIK3CA(H1047R) and pLenti-PIK3CA(E545K) constructs were subcloned into pLenti vector by the Gateway PCR cloning strategy (Invitrogen). Plasmids were purified using the Endotoxin free Maxi prep kit (Sigma-Aldrich, St. Louis, MO) for in vivo experiments.

### Hydrodynamic injection and mouse treatment

*Sgk3*^*+/−*^ mice were kindly provided by Dr. David Pearce from UCSF [26]. *Sgk3*^*+/−*^mice were bred together to generate *Sgk3* knockout mice; and *Sgk3*^*+/+*^ littermates were used as control. Hydrodynamic injection was performed using 5~7 week old mice as described previously [27]. In brief, to determine whether overexpression of PIK3CA plasmid alone can induce hepatic steatosis and carcinogenesis, 20 μg pT3-EF1α-PIK3CA(H1047R) or pT3-EF1α-PIK3CA(E545K) along with 0.8 μg pCMV/SB plasmid were diluted in 2 mL saline (0.9% NaCl) for each mouse. Mice were harvested 4 weeks post injection by Isoflurane inhalation followed by cervical dislocation. For the tumorigenesis models, 20 μg pT3-EF1α-PIK3CA(H1047R), or pT3-EF1α-PIK3CA(E545K) or PX330-sgPten were mixed with 20 μg pT3-EF1α-c-Met and 1.6 μg pCMV/SB and diluted in 2 mL saline (0.9% NaCl) for each mouse. Saline solution was filtered through a 0.22 μm filter and injected into the lateral tail vein of *Sgk3*^*+/+*^ or *Sgk3*^*−/−*^ mice within 5–7 s. Mice were monitored weekly and harvested when they developed swelling abdomen, which indicated large liver tumor burden based on UCSF IACUC protocol (number: AN173073).

### Histology, Immunohistochemistry, and Western blot analysis

Liver specimens were fixed overnight in zinc formalin (Anatech Ltd., Battle Creek, MI), embedded in paraffin, cut into 5-μm-thick sections, and placed on glass slides. Preneoplastic and neoplastic mouse liver lesions were evaluated by an experienced liver pathologist (S.R.) in accordance with the criteria described in detail previously [20, 28]. Imaging was performed with the automated Leica Bond^tm^ staining system (Leica Biosystems, Wetzlar, Germany). Frozen mouse liver specimens were homogenized in Mammalian Protein Extraction reagent (Thermo Scientific, Waltham, MA) containing the Complete Protease Inhibitor Cocktail and sonicated. Protein concentrations were determined with the Bio-Rad Protein Assay Kit (Bio-Rad, Hercules, CA), using bovine serum albumin as standard. Equal loading was assessed by GAPDH and β-actin. Immunohistochemistry (IHC) and Western blot analysis were performed as previously described [24, 29]. Primary antibodies for Immunohistochemistry and Western blotting are listed in Additional file 1: Table S1.

### Oil red O staining

Frozen sections of 10-μm were rehydrated, and lipid droplet deposition was detected by Oil Red O staining following the manufacturers’ instructions (American MasterTech, Lodi, CA, USA).

### In vitro studies

The human HuH7 and HLE HCC cell lines were used in this study. The source and other information of the cell lines were shown in Additional file 1: Table S2. Cells were grown in a 5% CO_2_ atmosphere, at 37 °C, in RPMI Medium supplemented with 10% fetal bovine serum (FBS; Gibco, Grand Island, NY, USA) and penicillin/streptomycin (Gibco). For silencing experiments, HuH7 and HLE cells were transfected with 50 nM small interfering RNA (siRNA) targeting human *SGK3* (ID # S24316; Life Technologies, Grand Island, NY) in the Lipofectamine RNAiMax Transfection Reagent (Life Technologies). A scramble siRNA (ID # 4390846; Life Technologies) was used as negative control. The AKT inhibitor, MK-2206 (Sigma-Aldrich; final concentration 2.5 μM), dissolved in DMSO, was administered to HuH7 and HLE cells for 24 and 48 h after 24 h serum deprivation, either alone or in association with siSGK3. Stable transfection experiments with pLenti-PIK3CA(H1047R) and pLenti-PIK3CA(E545K), respectively, were conducted in the two HCC cell lines. Before transfection, pLenti-PIK3CA(H1047R) and pLenti-PIK3CA(E545K) were packaged by 293 T cells to produce Lentivirus. When cells reached 50–60% confluency in 60 × 15 mm culture dishes, lentivirus was added into culture medium. 48–72 h later, cells were trypsinized and cultured in 100 × 20 mm culture dishes in culture medium containing puromycin at the concentration of 2μg/ml for both HuH7 and HLE. Cell proliferation and apoptosis were determined in human HCC cell lines at 24-, 48-, and 72-h time-points using the BrdU Cell Proliferation Assay Kit (Cell Signaling Technology Inc.) and the Cell Death Detection Elisa Plus Kit (Roche Molecular Biochemicals, Indianapolis, IN, USA), respectively, following the manufacturers’ instructions. All experiments were repeated at least three times in triplicate.

### Assessment of total cholesterol and triglyceride content

Total cholesterol and triglyceride levels in HuH7 and HLE cell lines were assessed using the Cholesterol Quantitation Kit and the Triglyceride Quantification Kit (BioVision Inc., Mountain View, CA, USA), respectively, following the manufacturer’s recommendation. All experiments were repeated at least three times in triplicate.

### Human liver specimens

A collection of formalin-fixed, paraffin-embedded HCC samples (*n* = 52) was used in the present study. Tumors were divided in HCC with shorter survival/poorer prognosis (HCCP; *n* = 28) and longer survival/better prognosis (HCCB; *n* = 24), characterized by < 3 and ≥ 3 years’ survival following partial liver resection, respectively. The clinicopathological features of liver cancer patients are summarized in Additional file 1: Table S3. Anonymized HCC specimens were generously provided by Dr. Snorri S. Thorgeirsson (National Institutes of Health, National Cancer Institute, Bethesda, MD) and collected at the University of Greifswald (Greifswald, Germany). Institutional Review Board approval was obtained at the National Institutes of Health and the local Ethical Committee of the Medical University of Greifswald (# BB 67/10) in compliance with the Helsinki Declaration. Written informed consent was obtained from all individuals. In these samples, immunohistochemical staining was performed on 10% formalin-fixed, paraffin-embedded sections, of human HCC specimens. Antigen retrieval was conducted in 10 mM citrate buffer (pH 6.0) by boiling the slides for 12 min in a pressure cooker, followed by a 30-min cool down at room temperature. Blocking was performed by incubating the slides with 5% goat serum and Avidin-Biotin blocking kit (Vector Laboratories, Burlingame, CA). Subsequently, the slides were incubated with the mouse monoclonal anti-SGK3 primary antibody (Santa Cruz Biotechnology Inc., Santa Cruz, CA; cat. N. sc-166,847; dilution 1:200) overnight at 4 °C. The following day, the endogenous peroxidase activity was suppressed by incubation of the slides in 3% hydrogen peroxide dissolved in methanol. Next, the biotin-conjugated secondary antibody was applied at a 1:500 dilution for 1 h at room temperature. The immunoreactivity was visualized with the Vectastain Elite ABC kit (Vector Laboratories, Burlingame, CA) and 3,3′- diaminobenzidine as the chromogen. Slides were counterstained with hematoxylin. Immunoreactivity for SGK3 was evaluated in a semi-quantitative manner: upregulation of SGK3 was defined when immunolabeling for SGK3 was stronger in tumors when compared to corresponding surrounding non-neoplastic livers.

### Statistical analysis

Data analysis was performed with Prism 6 Software (GraphPad, San Diego, CA). Differences between two groups were analyzed with unpaired t test. Kaplan–Meier method was used for survival analysis. *P* values < 0.05 were considered as statistically significant.

## Results

### Sgk3 deficiency does not affect PIK3CA mutant induced hepatic steatosis in mice

**Fig. 1:**
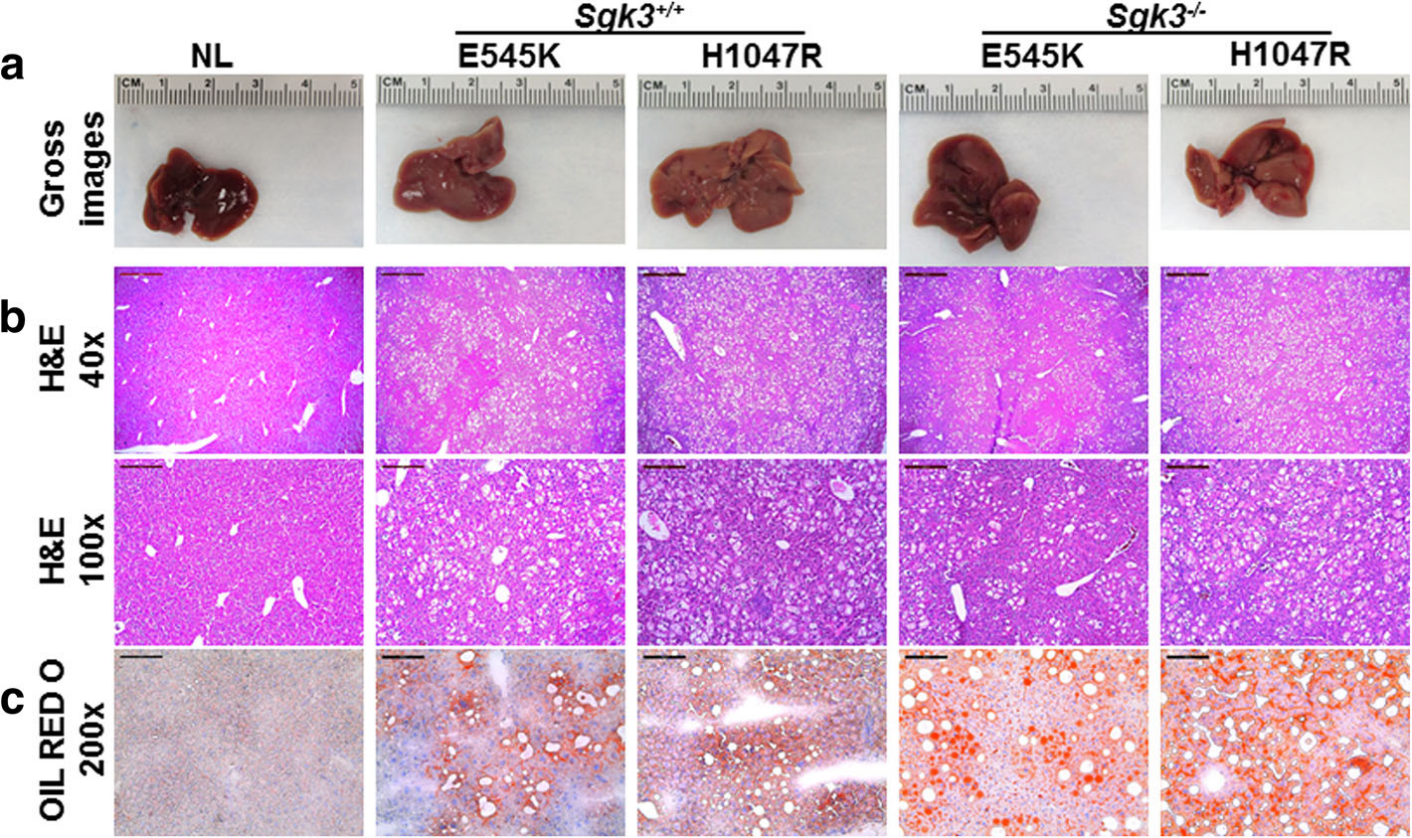
Overexpression of PIK3CA E545K or H1047R mutant induces hepatic steatosis in *Sgk3* knockout mice. **a** Gross image of livers from *Sgk3* wild-type (*Sgk3*^+/+^) and *Sgk3* knockout (*Sgk3*^−/−^) mice injected with PIK3CA(E545K) and PIK3CA(H1047R) constructs. Mice were sacrificed 4 weeks post hydrodynamic injection. **b** H&E staining of PIK3CA(E545K) and PIK3CA(H1047R) mouse livers; magnifications: 40x (scale bar = 500 μm) and 100x (scale bar = 200 μm). **c** ORO staining, magnifications: 200x. Abbreviation: H&E, haematoxylin and eosin staining; ORO, Oil Red O staining

Previously, we showed that activated mutant forms of PIK3CA alone induce hepatic steatosis when overexpressed in the mouse liver [20]. To determine whether SGK3 is required for activated PIK3CA mutant induced hepatic steatosis in vivo, we hydrodynamically transfected PIK3CA(H1047R) and PIK3CA(E545K) constructs, which we will refer here to as H1047R and E545K, into the *Sgk3*^*+/+*^ or *Sgk3*^*−/−*^ mouse liver. Mice were harvested 4 weeks post injection. Macroscopically, livers from all groups appeared to be pale and spotty (Fig. 1a). Histological examination revealed that both H1047R and E545K mouse livers in *Sgk3*^*+/+*^ or *Sgk3*^*−/−*^ genetic background showed the presence of numerous lipid-rich hepatocytes (Fig. 1b), leading to hepatic steatosis. The results were confirmed by Oil Red O (ORO) staining (Fig. 1c).

To further substantiate the in vivo findings, we silenced the *SGK3* gene in HuH7 and HLE human HCC cell lines using a validated combination of siRNA (Additional file 2: Figure S1). Of note, no changes in the major lipogenic proteins such as fatty acid synthase (FASN), acetyl-CoA carboxylase (ACC), and stearoyl-CoA desaturase 1 (SCD1) were detected by Western blot analysis between control and SGK3-depleted HuH7 and HLE cells. In addition, levels of activated/phosphorylated AKT, the master regulator of lipogenesis [20], were not affected by SGK3 suppression. Similarly, no evident changes in either triglycerides or cholesterol content accompanied *SGK3* silencing in the same cell lines (Additional file 2: Figure S1).

Altogether, the results demonstrate that loss of SGK3 does not affect activated H1047R or E545K PIK3CA mutants induced hepatic steatosis in mice and lipogenesis in HCC cell lines.

### Ablation of Sgk3 delays E545k/c-Met driven HCC development in mice

**Fig. 2:**
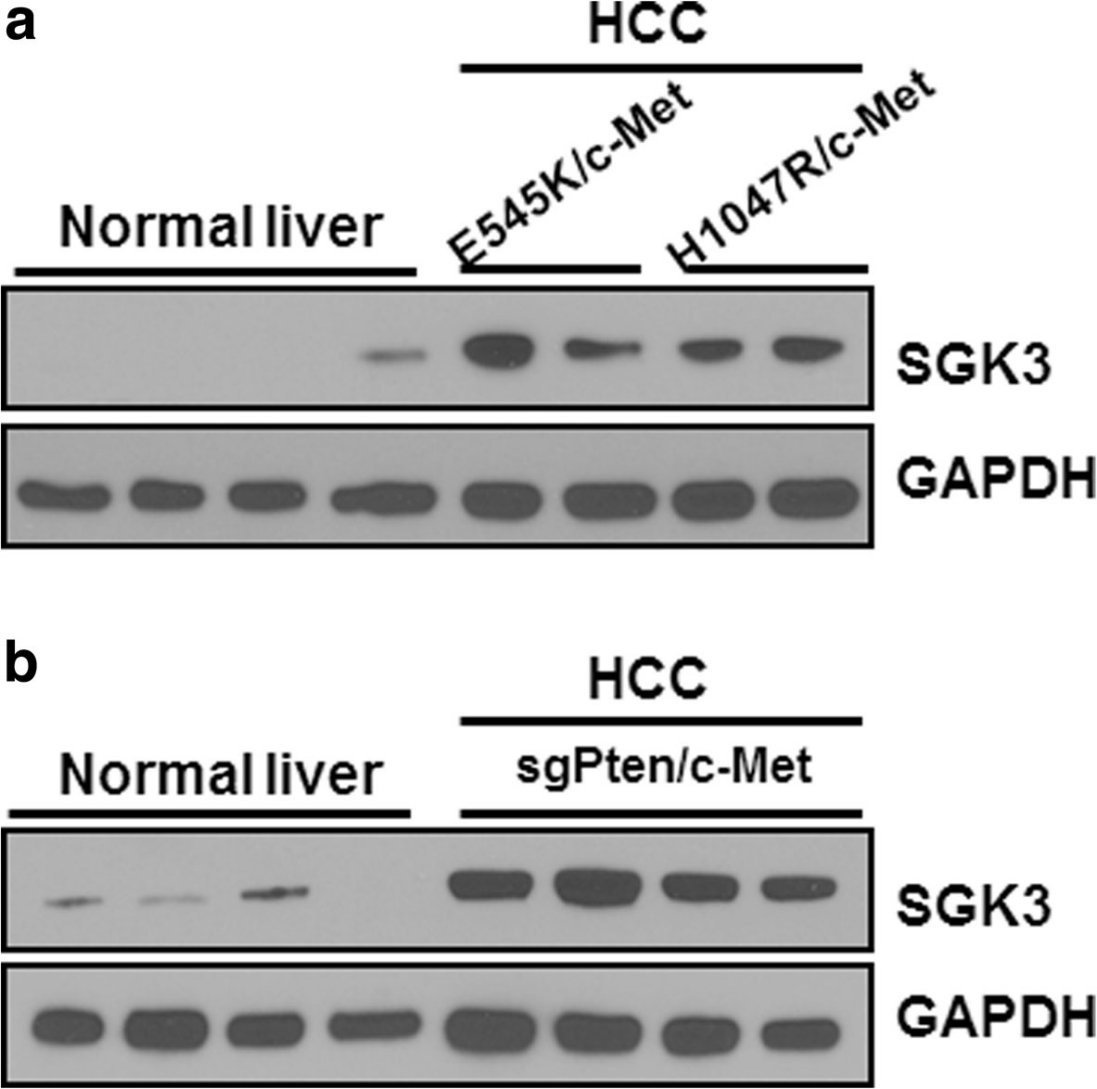
SGK3 expression is upregulated in E545K/c-Met, H1047R/c-Met and sgPten/c-Met HCC tissues. **a** SGK3 protein level in normal liver (wild-type) and PIK3CA/c-Met induced tumor samples by Western blotting. **b** SGK3 protein level in normal liver (wild-type) and sgPten/c-Met induced liver tumor samples by Western blotting. GAPDH was used as loading control

Next, we investigated whether SGK3 expression is required for PIK3CA mutants or loss of Pten induced HCC formation in mice. First, we determined SGK3 expression in E545K/c-Met, H1047R/c-Met, and sgPten/c-Met HCC tumor tissues. SGK3 protein expression was low in normal liver, and its levels increased in E545K/c-Met, H1047R/c-Met, and sgPten/c-Met HCC tissues (Fig. 2a and b).

**Fig. 3:**
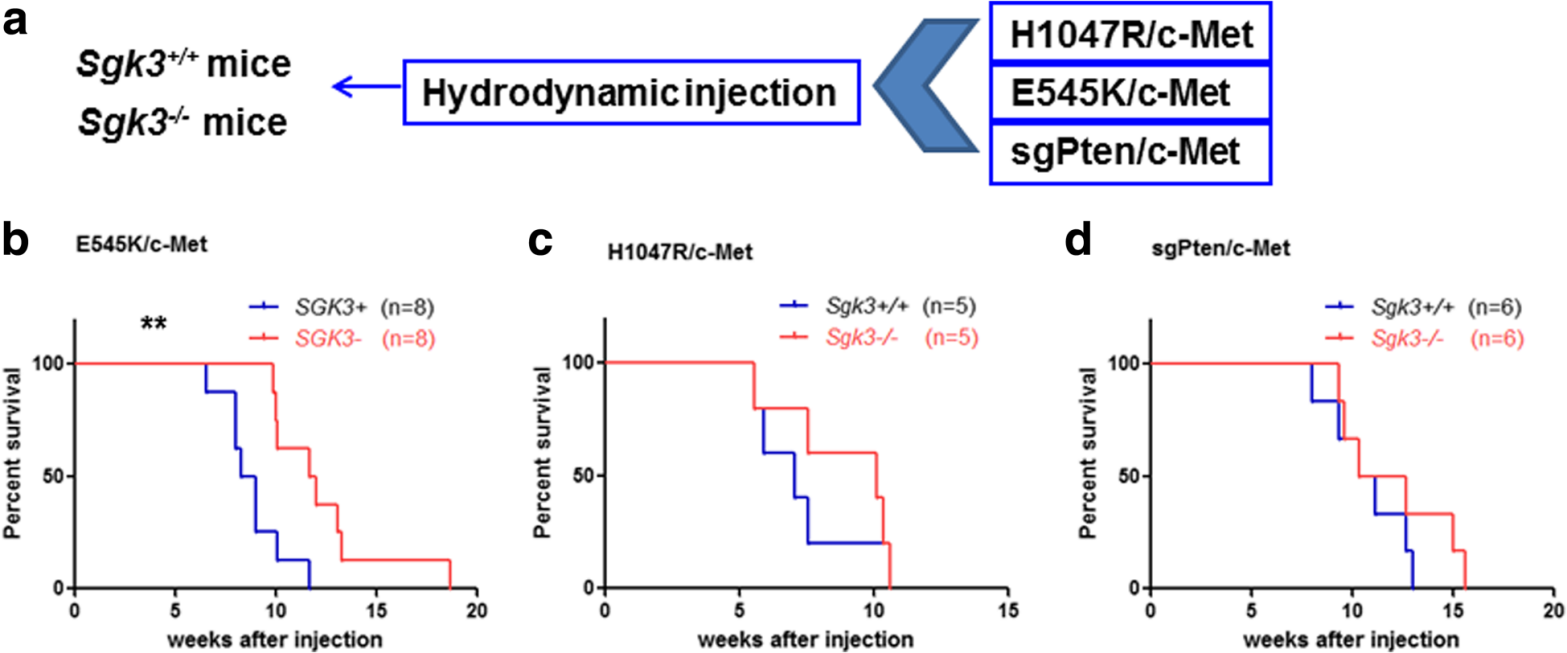
SGK3 is required for E545K/c-Met induced hepatocellular carcinoma. **a** Study design. **b** Survival curve of *Sgk3*^+/+^ and *Sgk*3^−/−^ mice bearing liver tumors by injection of E545K and c-Met constructs (**P = 0.0039; *n* = 8, respectively), **c** by injection of H1047R and c-Met constructs (*P* = 0.4635; *n* = 5, respectively), and **d** by injection of sgPten and c-Met constructs (*P* = 0.4732; *n* = 6, respectively)

In order to determine whether the ablation of SGK3 affects PIK3CA mutants or loss of Pten induced hepatocarcinogenesis in E545K/c-Met mice, we hydrodynamically injected PIK3CA(E545K) together with c-Met in *Sgk3*^*+/+*^ and *Sgk3*^*−/−*^ mice (Fig. 3a). E545K/c-Met injected *Sgk3*^*+/+*^ mice started to develop lethal burden of liver tumor by 6.6 to 11.7 weeks post injection and were required to be euthanized. In contrast, E545K/c-Met injected *Sgk3*^*−/−*^ mice developed lethal burden liver tumors by 9.9 to 18.7 weeks post injection (Fig. 3b, Table 1). The difference of survival rate was statistically significant (*p* = 0.0039, Fig. 3b). In striking contrast, no difference in the survival rate was detected between *Sgk3* wild-type and knockout mice after injection of H1047R/c-Met (Fig. 3c, Table 1) or sgPten/c-Met (Fig. 3d, Table 1).

**Table 1:** Detailed Mouse Data from E545K, H1047R or SgPten Combined with c-Met Injected Mice

Injection	*Sgk3* genotype	Number	Gender	Weeks post injection	Body weight (g)	Liver weight (g)	Histology
E545K/c-Met	+/+	8	F	6.6	23.7	5.8	HCC
			F	8	24.2	4.7	HCC
			F	8	25.3	5.6	HCC
			F	8.3	27.4	5.5	HCC
			M	9	32.5	7.4	HCC
			F	9.0	26.3	5.0	HCC
			M	10.1	31.0	7.1	HCC
			M	11.7	31.2	6.3	HCC
	−/−	8	F	9.9	26.9	5.3	HCC
			F	10.0	26.5	5.2	HCC
			M	10.1	35.4	7.4	HCC
			F	11.7	26.4	5.4	HCC
			M	12	29.3	6.8	HCC
			M	13.1	29.9	7.7	HCC
			M	13.3	32.8	7.0	HCC
			M	18.7	31.2	5.9	HCC
H1047R/c-Met	+/+	5	F	5.6	26.9	5.0	HCC
			F	5.9	21.3	5.9	HCC
			M	7.1	29.3	6.4	HCC
			M	7.6	31.3	5.9	HCC
			M	10.6	27.8	5.0	HCC
	−/−	5	F	5.6	28.0	6.4	HCC
			F	7.6	23.0	6.1	HCC
			M	10.1	31.0	6.0	HCC
			M	10.4	30.0	6.3	HCC
			M	10.6	28.2	6.1	HCC
sgPten/c-Met	+/+	6	F	8	23.1	5.3	HCC
			F	9.4	27.3	5.8	HCC
			M	10.4	29.2	6.3	HCC
			M	10.4	27.9	4.3	HCC
			M	12.7	31.8	6.8	HCC
			M	12.7	29.6	6.1	HCC
sgPten/c-Met	−/−	6	F	9.4	28.2	6.9	HCC
			M	9.6	27.5	7.3	HCC
			F	10.4	27.2	7.5	HCC
			F	12.7	26.8	5.1	HCC
			F	15	27.8	7.0	HCC
			M	15.6	29.2	8.1	HCC

**Fig. 4:**
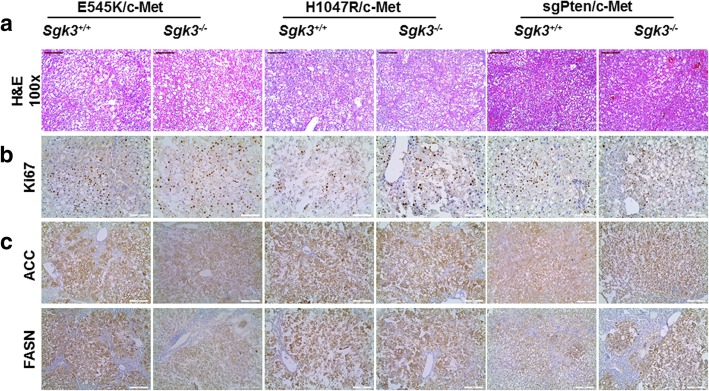
Histological staining of liver tumor in *Sgk3*^+/+^ and *Sgk3*^−/−^ mice injected with E545K, H1047R, or sgPten and c-Met constructs. **a** H&E staining of livers from *Sgk3*^+/+^ or *Sgk3*^−/−^ mice injected with oncogenic constructs, magnification: 100x, scale bar = 200 μm. **b** Representative immunohistochemical staining of ki67; original magnification: 200x, scale bar = 100 μm. **c** Representative immunohistochemical staining of FASN and ACC lipogenic proteins, original magnification 100x, scale bar = 200 μm

At the microscopic level, all tumors in *Sgk3*^*+/+*^ or *Sgk3*^*−/−*^ genetic background showed equivalent histological features. Indeed, the liver parenchyma was occupied by well to moderately differentiated HCC lesions, and tumors exhibited mostly a clear cell phenotype. No cholangiocarcinoma lesions were detected (Fig. 4a).

At the cellular level, tumor cells were highly proliferative, as visualized by Ki-67 immunostaining (Fig. 4b). Upon quantification, there was no statistical difference in the percentage of Ki-67 positive cells in any of the HCC models between *Sgk3*^*+/+*^ and *Sgk3*^*−/−*^ mice (Additional file 2: Figure S2). Furthermore, consistent with increased fat accumulation observed histologically, increased expression of lipogenesis genes, including FASN and ACC, could be readily detected by immunostaining in all HCC lesions (Fig. 4c).

**Fig. 5:**
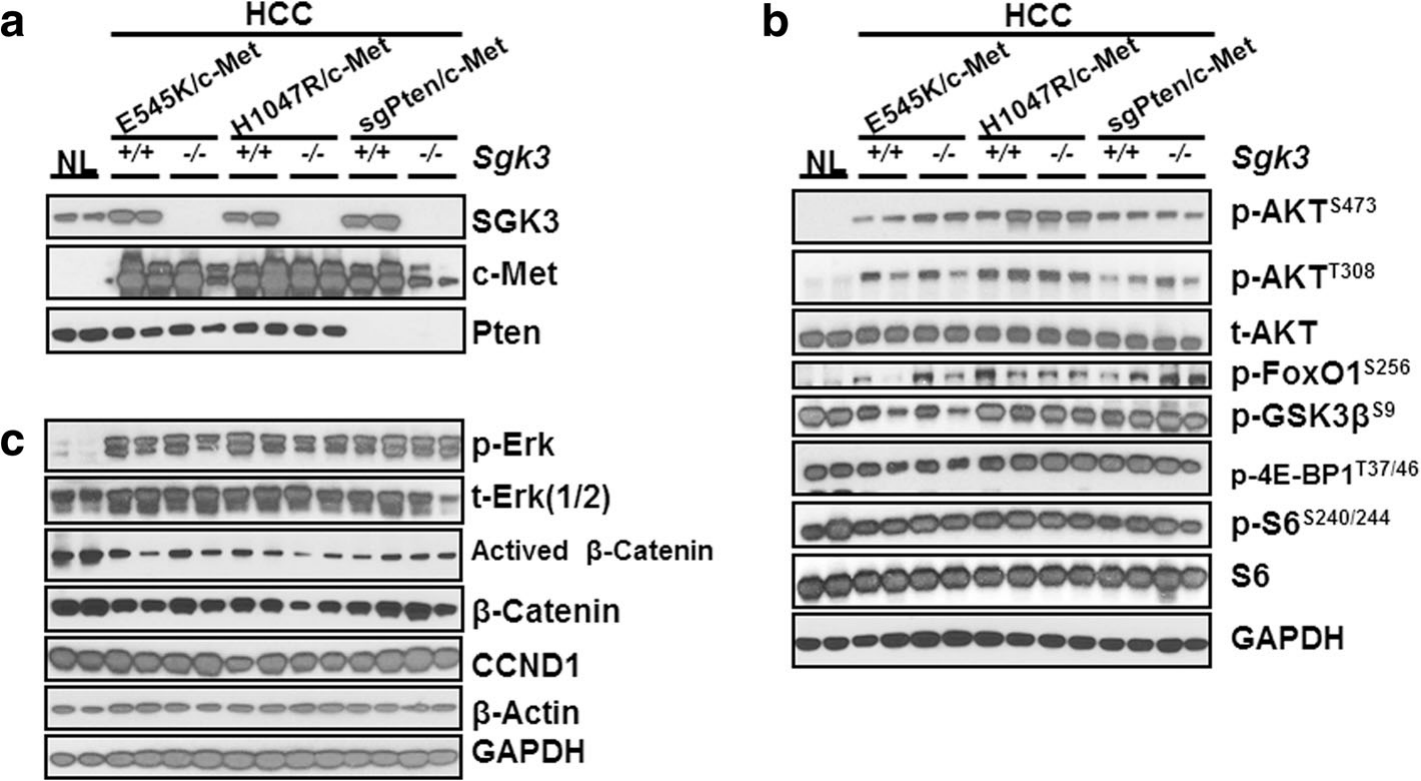
Molecular characterization of hepatocellular carcinoma developed in *Sgk3*^+/+^ and *Sgk3*^−/−^ mice injected with E545K, H1047R, or sgPten and c-Met constructs. Levels of SGK3, c-Met, Pten (**a**) activation of AKT/mTOR, (**b**) Ras/MAPK pathways, and β-Catenin (**c**) were assessed by Western blot analysis. Representative images are shown. GAPDH and β-actin were used as loading control. For detail description, please refer to the main text

At the molecular level, using Western blot analysis, we found that SGK3 was not expressed in HCCs from *Sgk3*^*−/−*^ mice (Fig. 5a). Human c-Met was found to be expressed in all tumor samples, while Pten protein expression was absent in sgPten/c-Met HCCs (Fig. 5a). Intriguingly, the levels of activated/phosphorylated AKT, (p-AKT^S473^) were lower in E545K/c-Met HCC from *Sgk3*^*+/+*^ mice than those from *Sgk3*^*−/−*^ mice (Fig. 5b); whereas p-AKT^T308^ was consistent in all HCC samples tested. For the downstream effectors of AKT/SGK, we found that p-FoxO1 expression was also higher in E545K/c-Met HCC from *Sgk3*^*−/−*^ mice (Fig. 5b), whereas other effectors, such as the mTOR cascade, indicated by surrogate markers of activation such as p-S6 or p-4EBP1, as well as p-GSK3β, showed similar expression in HCCs from *Sgk3*^*−/−*^ mice and *Sgk3*^*+/+*^ mice (Fig. 5b). In addition, loss of *Sgk3* did not affect ERK activation (Fig. 5c).

**Fig. 6:**
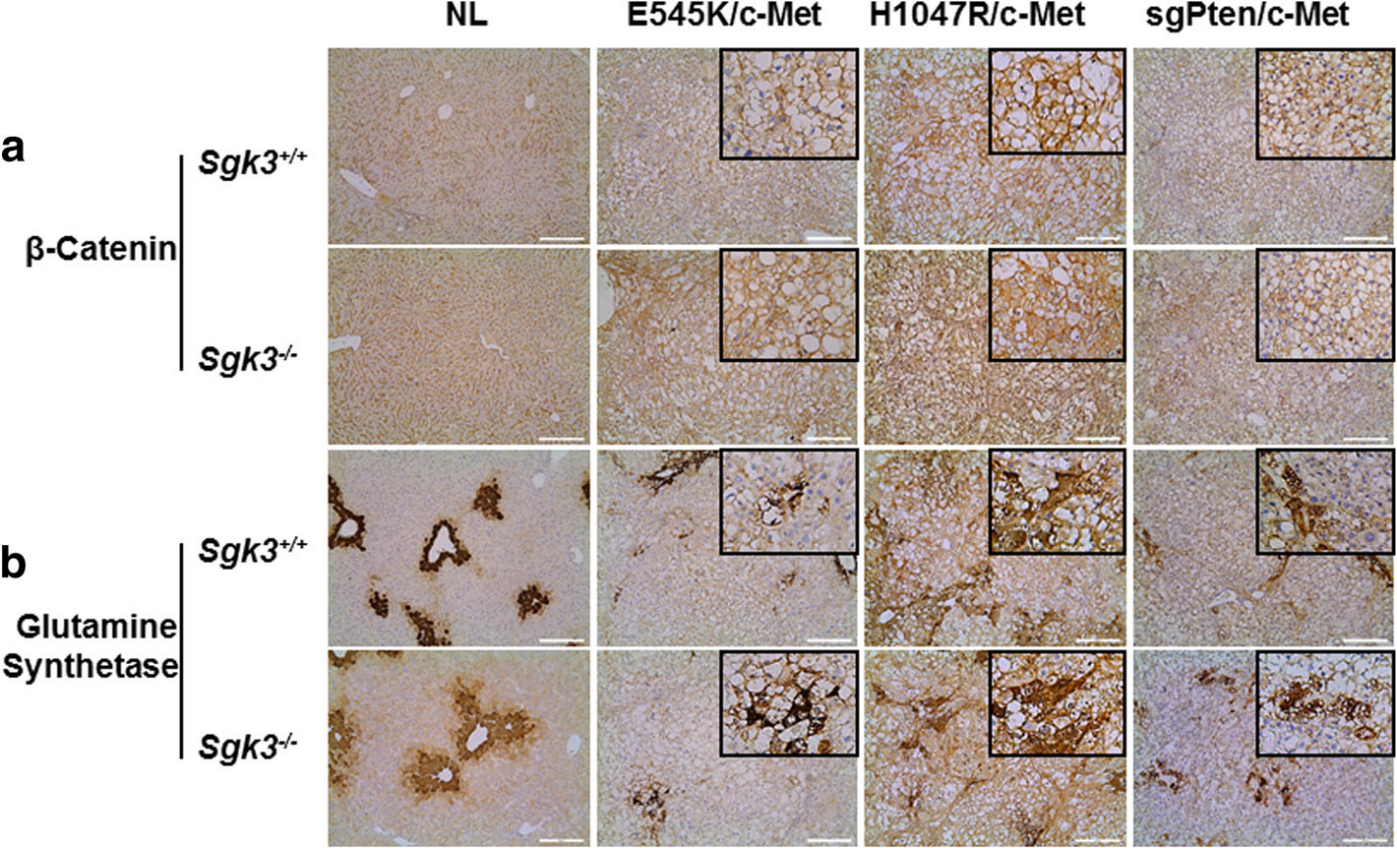
Immunohistochemical staining of β-Catenin (**a**) and Glutamine Synthetase (GS) (**b**) in *Sgk3*^+/+^ and *Sgk3*^−/−^ mice injected with E545K, H1047R, or sgPten and c-Met constructs. Original magnification: 100x, scale bar = 200 μm; × 200 (insets). For detailed description, please refer to the main text

**Fig. 7:**
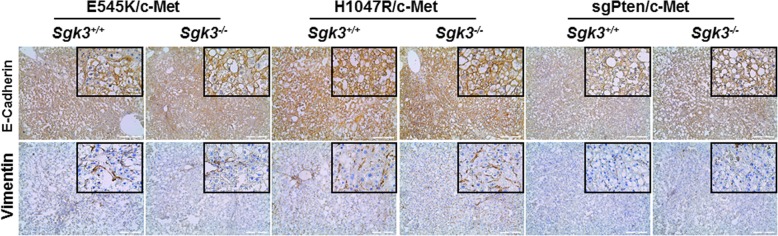
Immunohistochemical staining of E-Cadherin and Vimentin in *Sgk3*^+/+^ and *Sgk3*^−/−^ mice injected with E545K, H1047R or sgPten and c-Met constructs. Original magnification: 100x, scale bar = 200 μm; × 200 (insets). For detailed description, please refer to the main text

In several studies, it has been described that SGK3 and its homologue SGK2 may regulate cyclin D1 expression, GSK3-β/β-catenin cascade as well as epithelial-mesenchymal transition (EMT) [15, 30–32]. Therefore, we analyzed these genes and pathways in HCCs from *Sgk3* wild-type and KO mice. Using Western blotting, we found that all tumors expressed cyclin D1 at comparable levels, irrespective of *Sgk3* status (Fig. 5c). Similarly, total β-catenin levels as well as the activated forms of β-catenin did not change significantly in HCCs from *Sgk3* wild-type or KO mice (Fig. 5c). Using IHC, we observed that β-catenin was expressed predominantly at the tumor cell membrane with some weak cytoplasmic staining in all tumor models tested (Fig. 6a). Consistently, glutamine synthetase (GS), a well-characterized liver specific marker of activated β-catenin [33], showed mainly a para-tumorous staining pattern. However, clusters of GS(+) HCC cells could be found in tumor nodules (Fig. 6b). These results indicate a weak activation of Wnt/β-catenin in these HCCs. As concerns EMT, we analyzed the expression patterns of E-cadherin and Vimentin using IHC. We found that all tumor cells exhibited membranous E-cadherin staining, and Vimentin could only be found in stromal cells, but not HCC cells, of either *Sgk3* wild-type or KO mice (Fig. 7). The data suggest that SGK3 does not play a critical role in regulating EMT, at least in these mouse HCC models.

In summary, our study demonstrates that ablation of *Sgk3* delays E545K/c-Met driven HCC formation in mice, and it has no effect on H1047R/c-Met or sgPten/c-Met induced HCC development. Furthermore, SGK3 does not appear to regulate Wnt/β-catenin, EMT, or cyclin D1 expression in vivo.

### SGK3 suppression is detrimental for the growth of E545K- but not H1047R-mutant human HCC cell lines

**Fig. 8:**
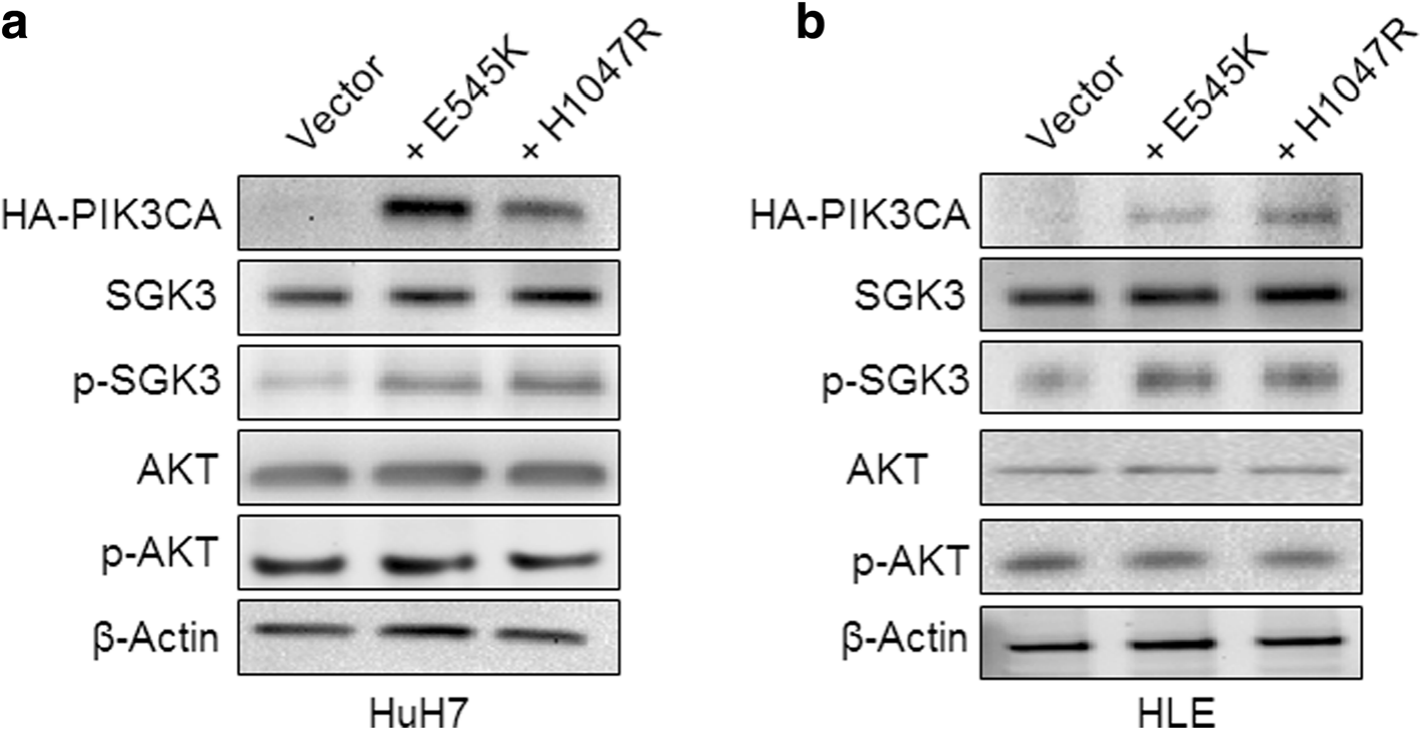
Generation of HuH7 (**a**) and HLE (**b**) human HCC cell lines stably expressing the PIK3CA E545K and H1047R mutants. Of note, stable transfection of the two PIK3CA mutants (+ E545K and + H1047R) resulted in a slight increase of SGK3 activation, but not AKT activation, in the two HCC cell lines. β-actin was used as loading control. Abbreviation: Vector, empty vector

**Fig. 9:**
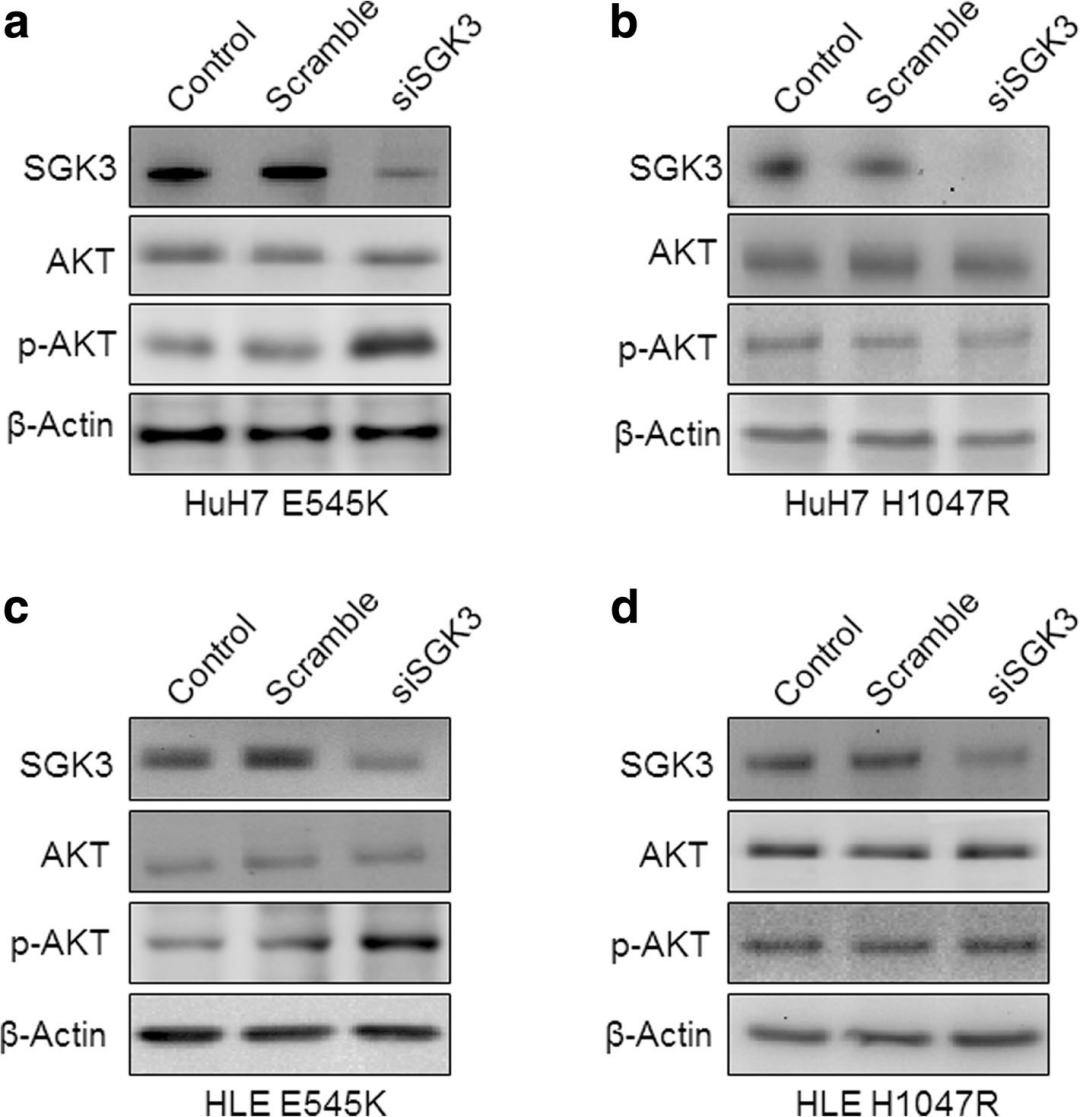
Silencing of SGK3 by siRNA triggers upregulation of activated/phosphorylated AKT in HUH7 (**a** and **b**) and HLE (**c** and **d**) HCC cell lines stably transfected with E545K (**a** and **c**) but not H1047R (**b** and **d**) mutant. Abbreviations: Scramble, scramble siRNA; siSGK3, siRNA against SGK3

Next, we assessed the importance of SGK3 on the growth of human HCC cell lines carrying E545K and H1047R mutations. Since, to the best of our knowledge, no HCC cell lines harbor PIK3CA mutations, we stably transfected the HuH7 and HLE cells with the E545K and H1047R mutants. Of note, forced overexpression of the two mutants led to slight induction of phosphorylation/activation of SGK3 but not AKT (Fig. 8). Subsequently, *SGK3* was silenced in HuH7 and HLE cells transfected with empty vector, E545K, or H1047R using specific siRNAs (Fig. 9). At the molecular level, suppression of *SGK3* triggered an induction of p-AKT in HuH7 and HLE cells transfected with the E545K but not H0147 mutant, when compared with the same cells transfected with the empty vector (Fig. 9). At the cellular level, overexpression of the two PIK3CA mutant forms resulted in a slight, equivalent increase of proliferation and decrease of apoptosis when compared with parental lines (Additional file 2: Figure S3). Importantly, suppression of *SGK3* by siRNA had minimal effect on the growth of vector- and H1047R-transfected HuH7 and HLE cells (Additional file 2: Figure S4). In striking contrast, a remarkable reduction in growth proliferation and elevated apoptosis was induced following SGK3 silencing in the two cell lines stably overexpressing E545K (Additional file 2: Figure S4).

Then, we evaluated whether inhibition of AKT (using the AKT inhibitor, MK-2206) acts synergistically with suppression of SGK3 (by siRNA) to constrain the growth of HuH7 and HLE transfected with the two PIK3CA mutants. Noticeably, a strong, synergistic anti-growth effect was achieved by the combination therapy only in HuH7 and HLE cells transfected with E545K (Additional file 2: Figure S5). Taken together, the data underline the importance of SGK3 downstream of the E545K mutant in HCC cells.

### SGK3 levels are increased in a human HCC subset with poor prognosis

**Fig. 10:**
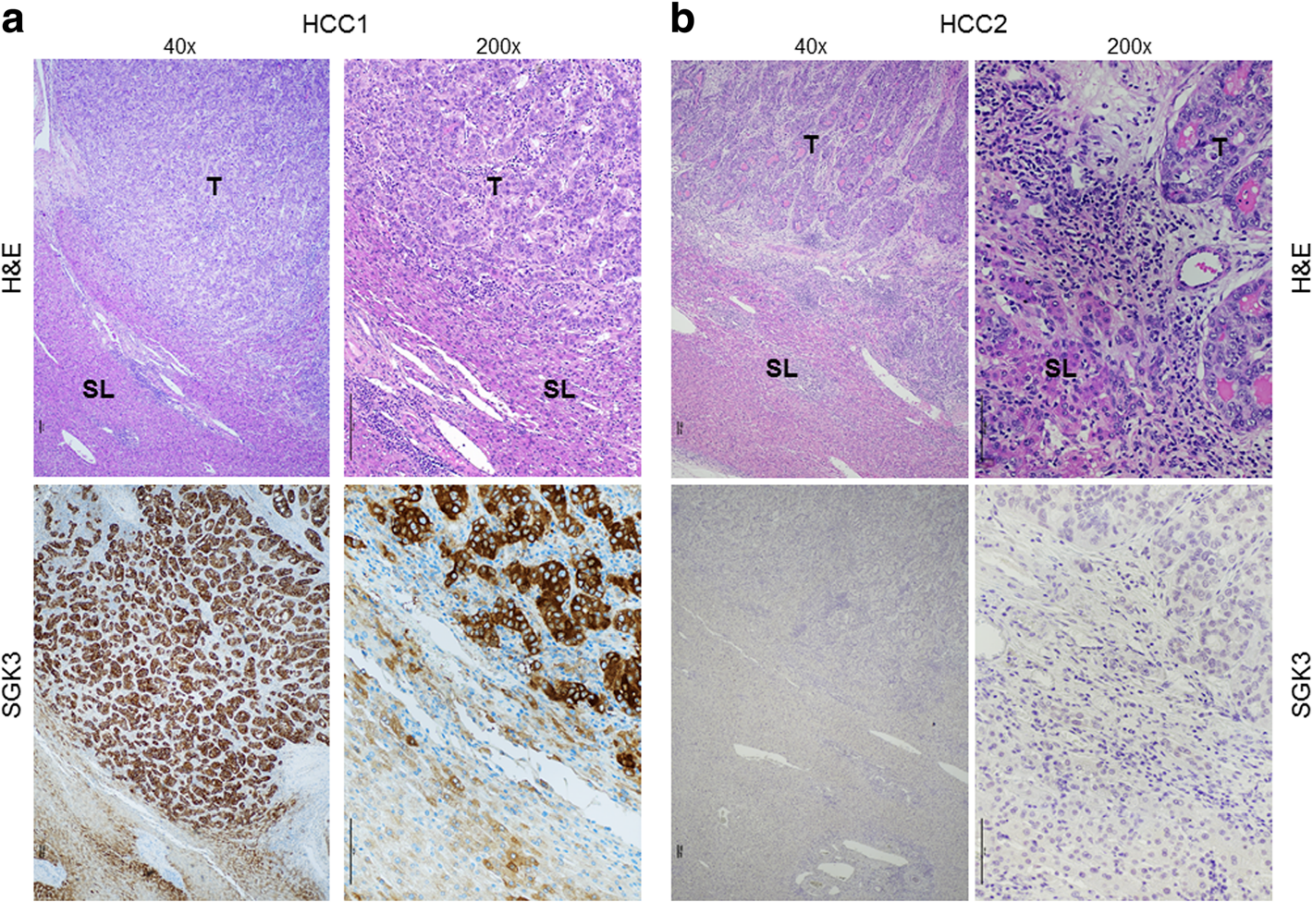
Immunohistochemical patterns of the SGK3 protein in human hepatocellular carcinoma (HCC). **a** Trabecular, well-differentiated HCC (HCC1) showing stronger cytoplasmic immunoreactivity for SGK3 in the tumorous part (T) than in the adjacent surrounding liver tissue (SL). **b** Moderately−/well-differentiated HCC sample (HCC2) exhibiting very low/absent immunoreactivity for SGK3 both in the tumorous and non-tumorous tissue. Scale bar: 100 μm: Magnifications: 40x and 200x. Abbreviation: H&E, hematoxylin and eosin staining

Finally, we analyzed SGK3 protein levels by Immunohistochemistry in a collection of human HCC samples (*n* = 52; Additional file 1: Table S3). Interestingly, we found that a subset of human HCC (20/52; 38.5%) exhibited higher SGK3 immunoreactivity in tumor when compared with the corresponding non-tumorous counterpart. The remaining samples (32/52; 61.5%) did not show significant staining differences between liver surrounding non-neoplastic and tumorous tissues, which often display absent or weak SGK3 immunostaining (Fig. 10). Of note, the vast majority (15/20; 75%) of the HCC displaying increased SGK3 immunolabeling in the tumor part belonged to the group associated with poorer prognosis (HCCP), suggesting that SGK3 expression might contribute to HCC aggressiveness and survival, in agreement with a previous study [15]. No association between the degree of SGK3 immunoreactivity and clinicopathologic features of the patients, including age, gender, etiology, presence of cirrhosis, tumor size, and tumor differentiation, was detected (data not shown).

## Discussion

SGK3 shares high homology with the AKT family and might have similar functions to AKT proteins in carcinogenesis. Emerging evidence indicates that SGK3 is critical for tumor cells’ survival, proliferation, and invasion [34]. Thus, it is not surprising that SGK3 has been found to be upregulated in a variety of human tumors, including HCC [15], breast cancer [35], and colon cancer [36]. In addition, silencing of *SGK3* has been proven to inhibit the growth of prostate cancer [13], HCC [15], melanoma [11], and breast cancer cell lines [37]. However, virtually all the studies were carried out in vitro using cell culture systems. To the best of our knowledge, this is the first report to use *Sgk3* KO mice to study the requirement of SGK3 along oncogene driven tumorigenesis in vivo. PIK3CA induces lipogenesis in normal hepatocytes and this phenotype is not affected by the loss of Sgk3. In contrast, human HCC cells are proliferating. Silencing of SGK3 inhibited E545K overexpressing HCC cell growth. These results suggest that SGK3 has distinct roles in normal hepatocytes versus HCC cells downstream of activated *PIK3CA* mutants.

In the present investigation, we discovered that ablation of *Sgk3* delays E545K/c-Met induced HCC formation in vivo, while not affecting PI3K/c-Met or sgPten/c-Met driven hepatocarcinogenesis. Interestingly, we found that phosphorylated/activated AKT levels were lower in E545K/c-Met HCCs from *Sgk3*^*+/+*^ mice than *Sgk3*^*−/−*^ mice, suggesting that higher levels of AKT activation might compensate for the loss of SGK3 in E545K/c-Met HCCs. The results corroborate a previous study showing that SGK3 is required for the growth of PIK3CA E545K mutant breast cancer cells [14]. Similarly, our in vitro data (this study) indicate that SGK3 inactivation is detrimental for the growth of E545K mutant, but not wild-type or H1047R mutant, HCC cells. In our previous investigation, we demonstrated that loss of *Akt2*, the major AKT isoform in the liver, is sufficient to completely prevent HCC development induced by E545K/c-Met or H1047R/c-Met in mice, suggesting that AKT2, rather than SGK3, is the major and critical downstream effector of activated *PIK3CA* mutants required for HCC formation. However, it is important to underline that all these studies investigated the requirement of AKT2 or SGK3 along HCC initiation. Additional work is necessary to determine whether AKT2 and/or SGK3 are needed for tumor progression (that is, maintaining cancer growth once tumors are fully formed). These studies should be carried out using inducible system, i.e., deleting *Akt2* or *Sgk3* in the liver of tumor bearing mice. As observed in HCC cell lines (this study), it is likely that concomitant inhibition of AKT2 and SGK3 signaling may be required to effectively restrain the growth of HCCs with PIK3CA E545K mutants in vivo. In agreement with the hypothesis that SGK3 might be critical in HCC progression rather than in liver tumor onset, we found that SGK3 was mainly induced in a subgroup of human HCC characterized by clinical aggressiveness, presumably contributing to poor prognosis. Of note, similar data were obtained in a previous, independent investigation [15]. Nonetheless, although interesting, these preliminary findings should be further substantiated in larger cohorts of HCC patients. Furthermore, in depth investigations should be aimed at identifying the molecular mechanisms and related pathways whereby SGK3 contributes to hepatocarcinogenesis as well as to an unfavorable patients’ outcome in liver cancer. In this regard, previous studies have shown that SGKs might modulate tumor growth via regulating cyclin D1 expression, GSK3-β/β-catenin cascade as well as epithelial-mesenchymal transition (EMT) in HCC cells [15, 30–32]. In light of the previous findings, we performed detailed analysis of these genes and pathways in *Sgk3* wild-type and KO mice. Unexpectedly, we did not observe any evidence that loss of *Sgk3* affects cyclin D1 expression, Wnt/β-catenin status, and/or EMT in vivo. The reason for the discrepancy between our findings and the previous data [15, 30–32] remains poorly understood. However, it is critical to note that the in vitro cell culture system is rather artificial and the molecular events described in previous studies should have been validated in in vivo models.

At the molecular level, in addition, we found that SGK3 does not regulate lipogenesis either in vivo or in vitro, thus implying that the specific targets of SGK3 and AKT (AKT2) might differ quite substantially in liver cells. As AKT2 depletion strongly suppresses de novo lipid biosynthesis and *Fasn* ablation impairs E545K/c-Met or H1047R/c-Met in mice (Che L et al., manuscript in preparation), these data might explain the different anti-neoplastic effect of AKT2 and SGK3 on the growth of HCC with PIK3CA mutations. Additional studies are needed to identify the SGK3 specific targets in the liver.

## Conclusion

Taken together, our data suggest that SGK3 plays a role in transducing helical domain mutant PIK3CA signaling during liver tumor development.

## Additional files

Additional file 1: **Table S1:** Western blotting (WB) and Immunohistochemistry (IHC) antibody information. **Table S2:** Cell Line Information. **Table S3:** Clinicopathological features of HCC Patients. (PDF 123 kb)

Additional file 2: **Figure S1:** SGK3 does not regulate lipogenesis in human HCC cell lines. **Figure S2:** Quantification of Ki67 in Sgk3+/+ and Sgk3−/− mice injected with E545K, H1047R, or sgPten and c-Met constructs. **Figure S3:** Effect of the PIK3CA mutants on cell proliferation and apoptosis of HCC cell lines. **Figure S4:** Effect of silencing of SGK3 by siRNA on cell proliferation and apoptosis of HCC cell lines stably transfected with E545K or H1047R mutant. **Figure S5:** Effect of the AKT inhibitor MK-2206 on cell proliferation and apoptosis of HCC cell lines stably transfected with E545K or H1047R mutant. (PDF 1752 kb)

Additional file 3: ARRIVE checklist. (PDF 640 kb)

## Abbreviations

EMT: Epithelial-mesenchymal transition
HCC: Hepatocellular carcinoma
IHC: Immunohistochemistry
mTOR: mammalian target of rapamycin
PI3K: Phosphoinositide-3-Kinase
PIK3CA: PI3K catalytic subunit alpha
Pten: Phosphatase and tensin homolog
SGK3: Serum/glucocorticoid regulated kinase 3

## References

[1] Siegel RL, Miller KD, Jemal A (2016). Cancer statistics, 2016. CA Cancer J Clin.

[2] Llovet JM, Ricci S, Mazzaferro V, Hilgard P, Gane E, Blanc JF (2008). Sorafenib in advanced hepatocellular carcinoma. N Engl J Med.

[3] Bruix J, Qin S, Merle P, Granito A, Huang YH, Bodoky G (2017). Regorafenib for patients with hepatocellular carcinoma who progressed on sorafenib treatment (RESORCE): a randomised, double-blind, placebo-controlled, phase 3 trial. Lancet..

[4] Fruman DA, Rommel C (2014). PI3K and cancer: lessons, challenges and opportunities. Nat Rev Drug Discov.

[5] Thorpe LM, Yuzugullu H, Zhao JJ (2015). PI3K in cancer: divergent roles of isoforms, modes of activation and therapeutic targeting. Nat Rev Cancer.

[6] Matter MS, Decaens T, Andersen JB, Thorgeirsson SS (2014). Targeting the mTOR pathway in hepatocellular carcinoma: current state and future trends. J Hepatol.

[7] Polivka J, Janku F (2014). Molecular targets for cancer therapy in the PI3K/AKT/mTOR pathway. Pharmacol Ther.

[8] Li J, Yen C, Liaw D, Podsypanina K, Bose S, Wang SI (1997). PTEN, a putative protein tyrosine phosphatase gene mutated in human brain, breast, and prostate cancer. Science..

[9] Cancer Genome Atlas Research Network (2017). Electronic address wbe, Cancer genome atlas research N. Comprehensive and integrative genomic characterization of hepatocellular carcinoma. Cell..

[10] Bruhn MA, Pearson RB, Hannan RD, Sheppard KE (2010). Second AKT: the rise of SGK in cancer signalling. Growth factors (Chur, Switzerland).

[11] Scortegagna M, Lau E, Zhang T, Feng Y, Sereduk C, Yin H (2015). PDK1 and SGK3 contribute to the growth of BRAF-mutant melanomas and are potential therapeutic targets. Cancer Res.

[12] Lang F, Bohmer C, Palmada M, Seebohm G, Strutz-Seebohm N, Vallon V (2006). (Patho)physiological significance of the serum- and glucocorticoid-inducible kinase isoforms. Physiol Rev.

[13] Wang Yuanzhong, Zhou Dujin, Chen Shiuan (2014). SGK3 Is an Androgen-Inducible Kinase Promoting Prostate Cancer Cell Proliferation Through Activation of p70 S6 Kinase and Up-Regulation of Cyclin D1. Molecular Endocrinology.

[14] Gasser JA, Inuzuka H, Lau AW, Wei W, Beroukhim R, Toker A (2014). SGK3 mediates INPP4B-dependent PI3K signaling in breast cancer. Mol Cell.

[15] Liu M, Chen L, Chan TH, Wang J, Li Y, Li Y (2012). Serum and glucocorticoid kinase 3 at 8q13.1 promotes cell proliferation and survival in hepatocellular carcinoma. Hepatology..

[16] Liu F, Wu X, Jiang X, Qian Y, Gao J (2018). Prolonged inhibition of class I PI3K promotes liver cancer stem cell expansion by augmenting SGK3/GSK-3beta/beta-catenin signalling. J Exp Clin Cancer Res.

[17] Stemke-Hale K, Gonzalez-Angulo AM, Lluch A, Neve RM, Kuo WL, Davies M (2008). An integrative genomic and proteomic analysis of PIK3CA, PTEN, and AKT mutations in breast cancer. Cancer Res.

[18] Vasudevan KM, Barbie DA, Davies MA, Rabinovsky R, McNear CJ, Kim JJ (2009). AKT-independent signaling downstream of oncogenic PIK3CA mutations in human cancer. Cancer Cell.

[19] Takiar V, Ip CK, Gao M, Mills GB, Cheung LW (2017). Neomorphic mutations create therapeutic challenges in cancer. Oncogene..

[20] Wang C, Che L, Hu J, Zhang S, Jiang L, Latte G (2016). Activated mutant forms of PIK3CA cooperate with RasV12 or c-met to induce liver tumour formation in mice via AKT2/mTORC1 cascade. Liver Int.

[21] Xu Z, Hu J, Cao H, Pilo MG, Cigliano A, Shao Z (2018). Loss of Pten synergizes with c-met to promote hepatocellular carcinoma development via mTORC2 pathway. Exp Mol Med.

[22] Galicia VA, He L, Dang H, Kanel G, Vendryes C, French BA (2010). Expansion of hepatic tumor progenitor cells in Pten-null mice requires liver injury and is reversed by loss of AKT2. Gastroenterology..

[23] Li L, Che L, Tharp KM, Park HM, Pilo MG, Cao D (2016). Differential requirement for de novo lipogenesis in cholangiocarcinoma and hepatocellular carcinoma of mice and humans. Hepatology..

[24] Hu J, Che L, Li L, Pilo MG, Cigliano A, Ribback S (2016). Co-activation of AKT and c-met triggers rapid hepatocellular carcinoma development via the mTORC1/FASN pathway in mice. Sci Rep.

[25] Xue W, Chen S, Yin H, Tammela T, Papagiannakopoulos T, Joshi NS (2014). CRISPR-mediated direct mutation of cancer genes in the mouse liver. Nature..

[26] McCormick JA, Feng Y, Dawson K, Behne MJ, Yu B, Wang J (2004). Targeted disruption of the protein kinase SGK3/CISK impairs postnatal hair follicle development. Mol Biol Cell.

[27] Chen X, Calvisi DF (2014). Hydrodynamic transfection for generation of novel mouse models for liver cancer research. Am J Pathol.

[28] Delogu S, Wang C, Cigliano A, Utpatel K, Sini M, Longerich T (2015). SKP2 cooperates with N-Ras or AKT to induce liver tumor development in mice. Oncotarget..

[29] Calvisi DF, Wang C, Ho C, Ladu S, Lee SA, Mattu S (2011). Increased lipogenesis, induced by AKT-mTORC1-RPS6 signaling, promotes development of human hepatocellular carcinoma. Gastroenterology..

[30] Wu M, Huang C, Huang X, Liang R, Feng Y, Luo X (2017). MicroRNA-144-3p suppresses tumor growth and angiogenesis by targeting SGK3 in hepatocellular carcinoma. Oncol Rep.

[31] Liu J, Zhang G, Lv Y, Zhang X, Ying C, Yang S (2017). SGK2 promotes hepatocellular carcinoma progression and mediates GSK-3beta/beta-catenin signaling in HCC cells. Tumour biol.

[32] Kong X, Liu F, Gao J (2016). MiR-155 promotes epithelial-mesenchymal transition in hepatocellular carcinoma cells through the activation of PI3K/SGK3/beta-catenin signaling pathways. Oncotarget..

[33] Cadoret A, Ovejero C, Terris B, Souil E, Levy L, Lamers WH (2002). New targets of beta-catenin signaling in the liver are involved in the glutamine metabolism. Oncogene..

[34] Bruhn MA, Pearson RB, Hannan RD, Sheppard KE (2013). AKT-independent PI3-K signaling in cancer - emerging role for SGK3. Cancer Manag Res.

[35] Sun X, Liu X, Liu BO, Li S, Zhang D, Guo H (2016). Serum- and glucocorticoid-regulated protein kinase 3 overexpression promotes tumor development and aggression in breast cancer cells. Oncol Lett.

[36] Guo ST, Chi MN, Yang RH, Guo XY, Zan LK, Wang CY (2016). INPP4B is an oncogenic regulator in human colon cancer. Oncogene..

[37] Wang Y, Zhou D, Phung S, Masri S, Smith D, Chen S (2011). SGK3 is an estrogen-inducible kinase promoting estrogen-mediated survival of breast cancer cells. Molecular endocrinology (Baltimore, Md).

