# Supplementary material for: Functional role of SGK3 in PI3K/Pten driven liver tumor development
Source: BMC Cancer. 2019 Apr 11;19:343. doi: 10.1186/s12885-019-5551-2 (PMC6458829; doi:10.1186/s12885-019-5551-2)
Supplement: Supplementary file 1 — Table S1: Western blotting (WB) and Immunohistochemistry (IHC) antibody information. Table S2: Cell Line Information. Table S3: Clinicopathological features of HCC Patients. (PDF 123 kb) [file 12885_2019_5551_MOESM1_ESM.pdf]

Supplementary Table 1: Western blotting (WB) and Immunohistochemistry (IHC) antibody information

Antibody	Company	Catalog number	Dilution	Method
GAPDH	EMD Millpore	MAB374	1:10000	WB
SGK3	Cell signaling technology	8573	1:1000	WB
c-Met	Abcam	ab59884	1:100	WB
Pten	Cell signaling technology	9188	1:1000	WB
Phospho-AKT^{S473}	Cell signaling technology	3787	1:1000	WB
Phospho-AKT^{T308}	Cell signaling technology	13038	1:1000	WB
AKT	Cell signaling technology	9272	1:1000	WB
Phospho-4E-BP1	Cell signaling technology	2855	1:2000	WB
Phospho-RPS6	Cell signaling technology	5363	1:2000	WB
RPS6	Cell signaling technology	2217	1:2000	WB
Phospho-ERK	Cell signaling technology	4370	1:1000	WB
ERK	Cell signaling technology	9102	1:1000	WB
p-FoxO1	Cell signaling technology	84192	1:1000	WB
p-GSK3β	Cell signaling technology	9336	1:1000	WB
Active β-Catenin	Cell signaling technology	4270	1:1000	WB
β-Catenin	BD Biosciences	BD610153	1:2000	WB
CCND1	Abcam	ab134175	1:20000	WB
β-actin	Sigma-Aldrich	A5441	1:4500	WB
E-Cadherin	BD Biosciences	610181	1:500	IHC
Vimentin	Cell signaling technology	5741	1:100	IHC
β-Catenin	BD Biosciences	BD610153	1:400	IHC
Glutamine Synthetase	BD Biosciences	BD 610518	1:500	IHC
ACC	Cell Signaling Technology	3676	1:100	IHC
FASN	Cell Signaling Technology	3180	1:150	IHC
Ki67	Thermo Scientific	MA5-14520	1:150	IHC

Supplementary Table 2: Cell Line Information

Name	Source	Citation	Cat no.	Authentication test method/Time	test method for mycoplasma contamination
HuH7	JCRB Cell Bank	Pubmed: 6286115	JCRB0403	STR 3/7/2016	PCR
HLE	JCRB Cell Bank	PubMed: 52570	JCRB0404	STR 3/7/2016	PCR

Supplementary Table 3: Clinicopathological features of HCC Patients

Variables	Features	
	HCCB[a]	HCCP[b]
No. of patients	24	28
Male	18	18
Female	6	10
Age (Mean ± SD)	63.8 ± 10.2	66.0 ± 10.8
Etiology		
HBV	10	12
HCV	8	10
Ethanol	6	6
Cirrhosis		
+	18	20
-	6	8
Tumor size		
> 5 cm	16	18
< 5 cm	8	10
Edmondson and Steiner grade		
II	8	5
III	10	11
IV	6	12
Alpha-fetoprotein secretion		
> 300 ng/ml of serum	14	19
< 300 ng/ml of serum	10	9
Survival after partial liver resection (months) Means ± SD	63.4 ± 22.2	16.8 ± 12.2

[a]HCCB, HCC with better prognosis/longer survival (survival ≥3 years following partial liver resection).

[b]HCCP, HCC with poorer prognosis/shorter survival (survival <3 years following partial liver resection).
