# Supplementary material for: Functional role of SGK3 in PI3K/Pten driven liver tumor development
Source: BMC Cancer. 2019 Apr 11;19:343. doi: 10.1186/s12885-019-5551-2 (PMC6458829; doi:10.1186/s12885-019-5551-2)
Supplement: Supplementary file 2 — Figure S1: SGK3 does not regulate lipogenesis in human HCC cell lines. Figure S2: Quantification of Ki67 in Sgk3+/+ and Sgk3−/− mice injected with E545K, H1047R, or sgPten and c-Met constructs. Figure S3: Effect of the PIK3CA mutants on cell proliferation and apoptosis of HCC cell lines. Figure S4: Effect of silencing of SGK3 by siRNA on cell proliferation and apoptosis of HCC cell lines stably transfected with E545K or H1047R mutant. Figure S5: Effect of the AKT inhibitor MK-2206 on cell proliferation and apoptosis of HCC cell lines stably transfected with E545K or H1047R mutant. (PDF 1752 kb) [file 12885_2019_5551_MOESM2_ESM.pdf]

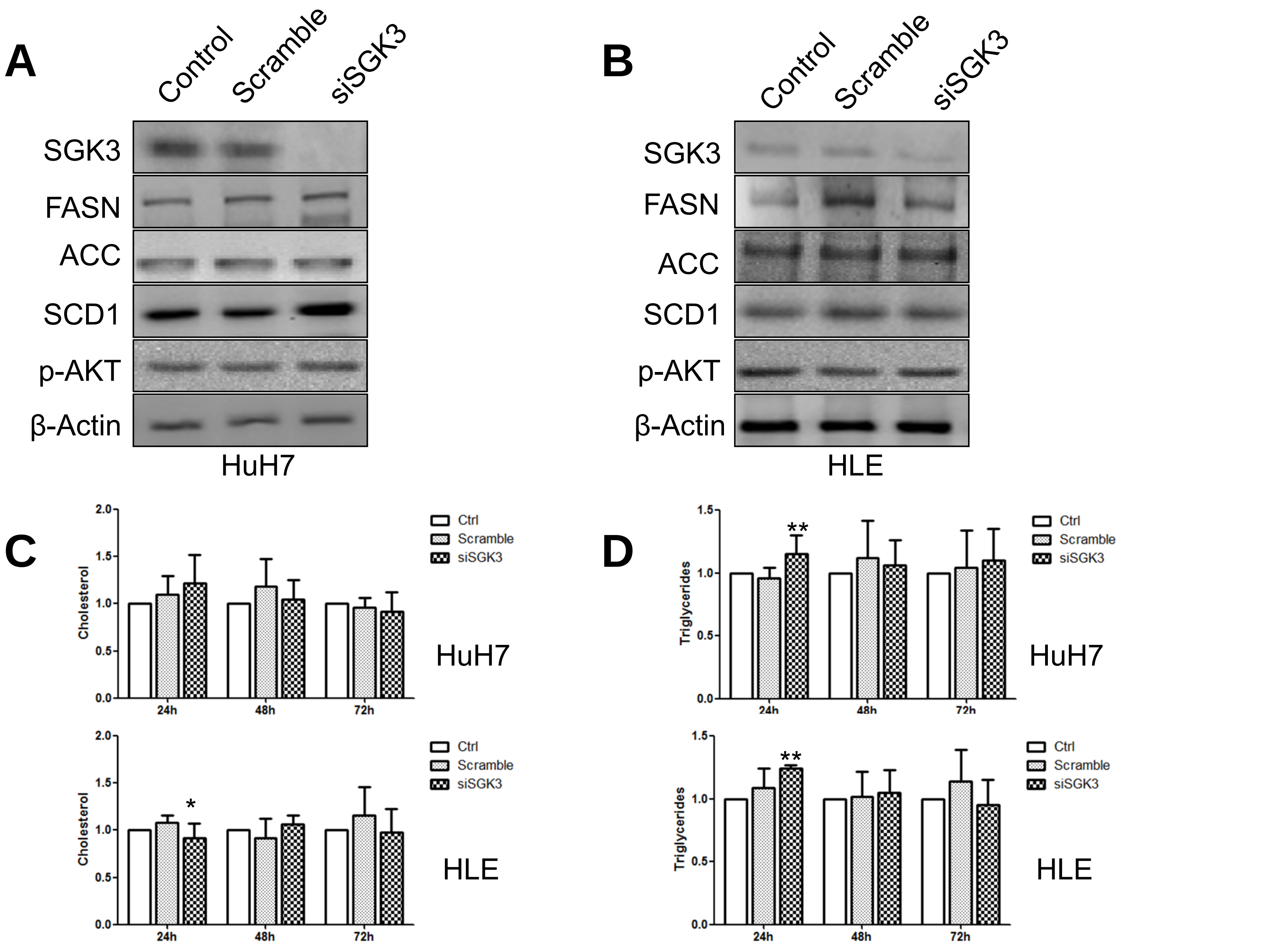

Supplementary Figure 1: SGK3 does not regulate lipogenesis in human HCC cell lines. (A) Suppression of *SGK3* expression by specific siRNA for 48h does not affect levels of FASN, ACC, and SCD1 lipogenic proteins and the master lipogenesis inducer activated/phosphorylated (p) AKT in HuH7 cells, as assessed by Western blot analysis. β-Actin was used as a loading control. **(B)** Equivalent results were obtained in HLE cells. **(C, D)** Knockdown of *SGK3* by siRNA for 24 h and 48h does not affect triglycerides and cholesterol levels in the two cell lines. Tukey-Kramer's test: * $P < 0.05$; ** $P < 0.01$.

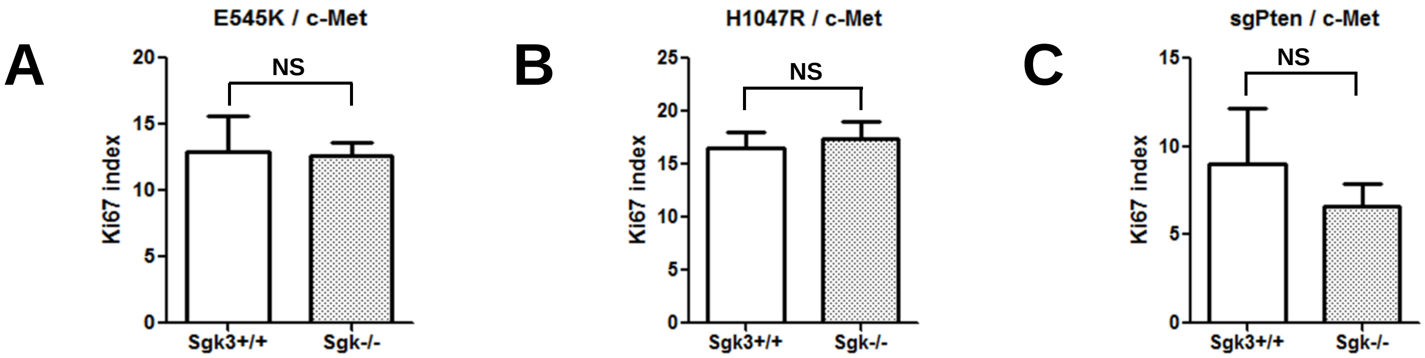

Supplementary Figure 2: Quantification of Ki67 in *Sgk3*$^{+/+}$ and *Sgk3*$^{-/-}$ mice injected with E545K, H1047R, or sgPten and c-Met constructs. (A) E545K/c-Met (*P* = 0.9198); **(B)** H1047R/c-Me (*P* = 0.6839); **(C)** sgPten/c-Met (*P* = 0.4951).

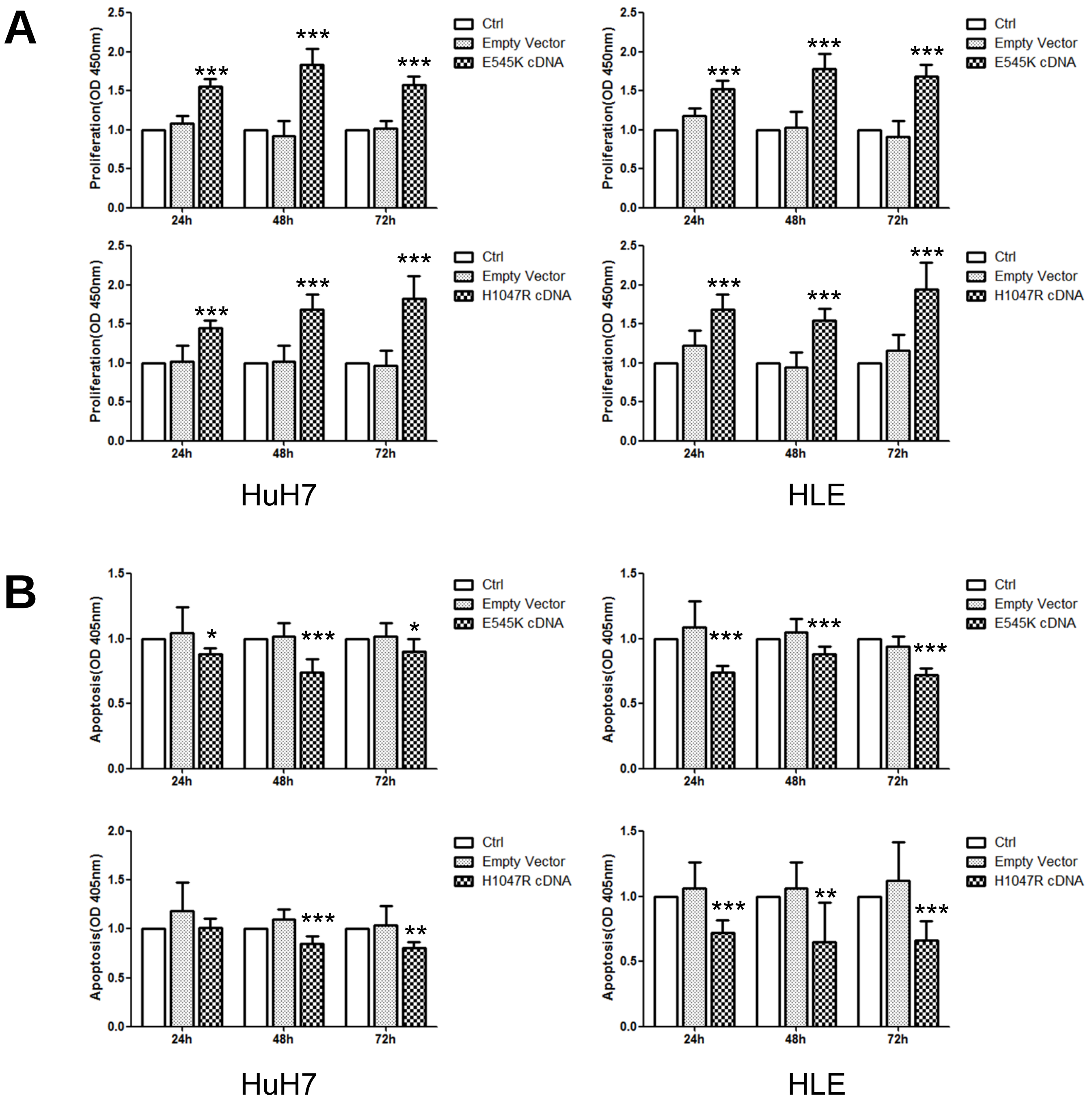

Supplementary Figure 3. Effect of the PIK3CA mutants on cell proliferation and apoptosis of HCC cell lines. (A) Cell proliferation in the HuH7 and HLE cell lines. (B) Apoptosis. Tukey-Kramer's test: * $P < 0.05$; ** $P < 0.01$; *** $P < 0.001$.

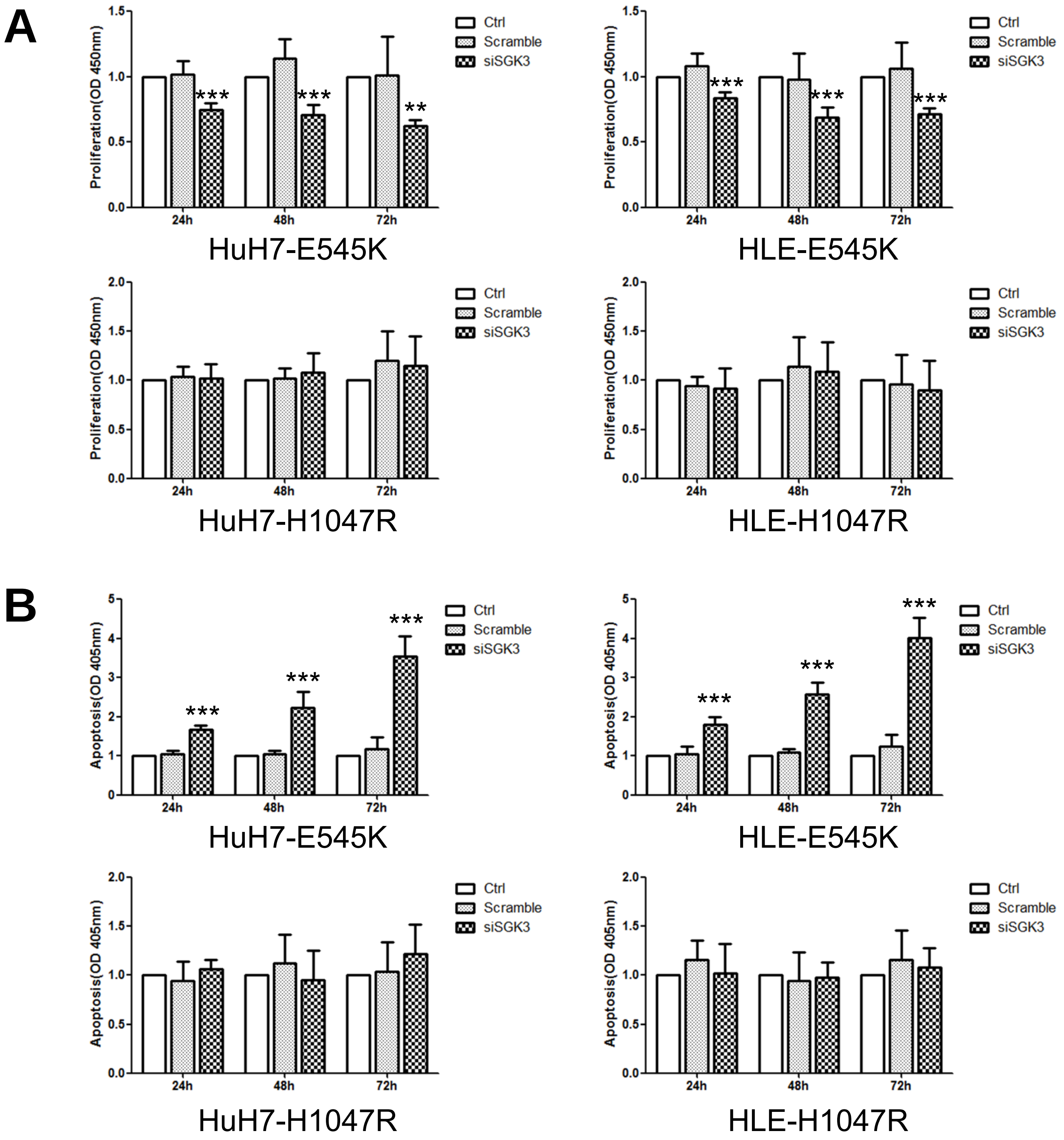

Supplementary Figure 4. Effect of silencing of SGK3 by siRNA on cell proliferation and apoptosis of HCC cell lines stably transfected with E545K or H1047R mutant. (A) Cell proliferation in the HuH7 and HLE cell lines stably transfected with E545K or H1047R mutant. (B) Apoptosis. Tukey-Kramer's test: * $P < 0.05$; ** $P < 0.01$; *** $P < 0.001$.

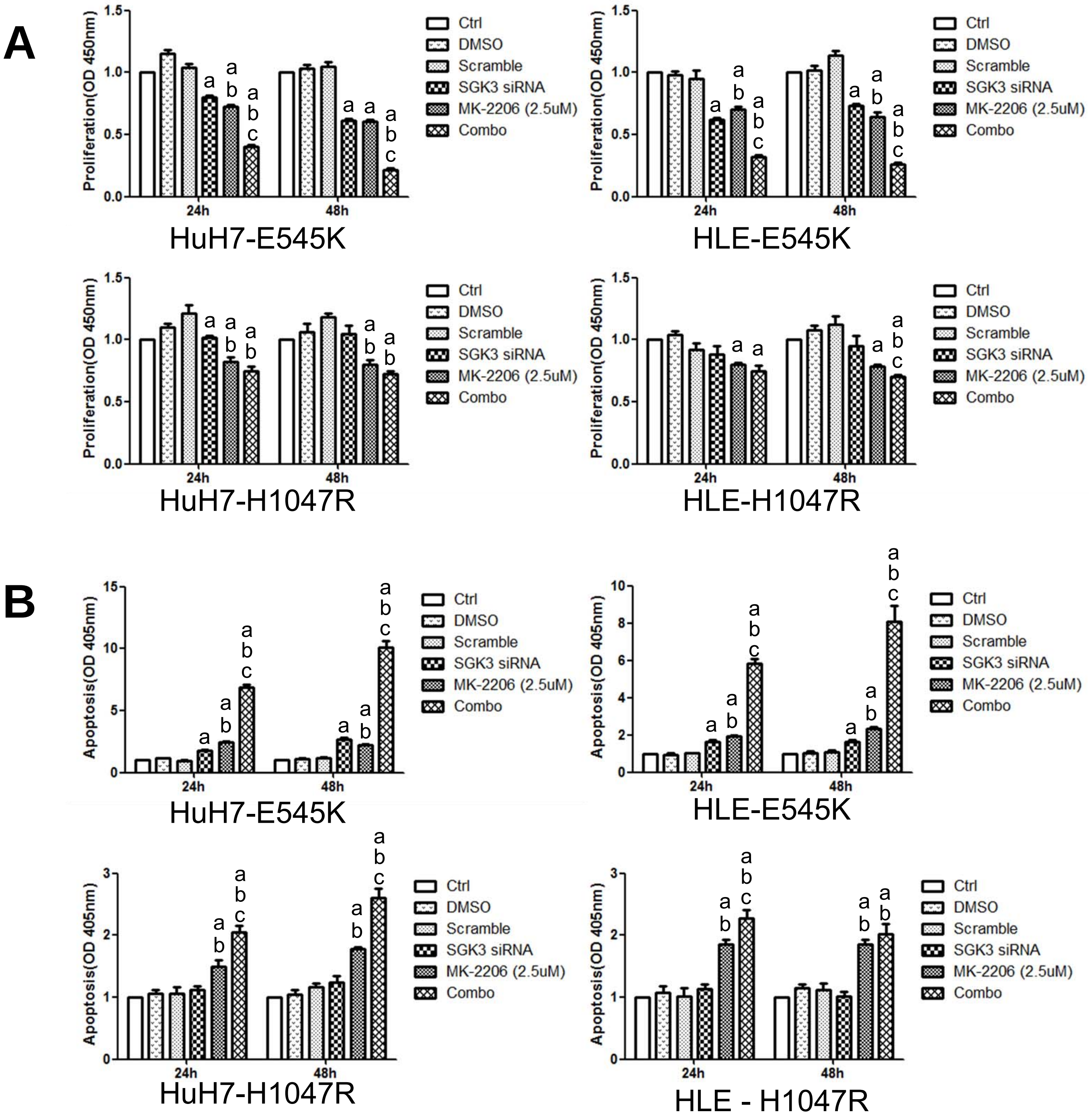

Supplementary Figure 5. Effect of AKT inhibitor MK-2206 on cell proliferation and apoptosis of HCC cell lines stably transfected with E545K or H1047R mutant. (A) Cell proliferation in the HuH7 and HLE cell lines stably transfected with E545K or H1047R mutant. (B) Apoptosis. Tukey Kramer test, *P* <0.05, a: vs Scrmble treated cells; b: vs SGK3 siRNA; c: vs MK-2206 (2.5 μm).
